# Biobased Organic Nanoparticles: A Promising Versatile Green Tool for Novel Antimicrobial Agents for Improved Safety

**DOI:** 10.1155/ijfo/7955106

**Published:** 2025-07-18

**Authors:** Edith Dube, Grace Emily Okuthe

**Affiliations:** Department of Biological & Environmental Sciences, Walter Sisulu University, Mthatha, South Africa

**Keywords:** cellular uptake, encapsulation, nanoparticle, naturally derived, pathogen

## Abstract

Biobased organic nanoparticles possess nanoscale size, large surface area, tunable surface charge, and strong functionalization capacity, significantly enhancing their antimicrobial performance. Derived from renewable materials such as polysaccharides, proteins, and plant-based compounds, they provide a sustainable and ecofriendly alternative to synthetic antimicrobial agents. Their antimicrobial effects include disrupting microbial membranes, generating reactive oxygen species, and interfering with essential cellular processes, making them effective even against drug-resistant pathogens. Additionally, when used as drug delivery systems, they improve therapeutic outcomes by enhancing compound stability, bioavailability, and targeted delivery while minimizing toxicity. This review comprehensively analyses their structural features, mechanisms of action, and dual roles in infection control and drug delivery. It also addresses key challenges, including nanoparticle stability, scalability, and biosafety. The review concludes with future directions, emphasizing the need to optimize nanoparticle design, understand their interactions with microbial cells, and develop safe, cost-effective, and environmentally friendly synthesis methods to support their broader application in sustainable healthcare.

## 1. Introduction

The global rise of antimicrobial resistance (AMR) represents a critical challenge to public health, threatening the effectiveness of current antimicrobial therapies and demanding the development of innovative and sustainable alternatives [1, 2]. In this context, nanoparticles (NPs) have garnered significant attention as next-generation antimicrobial agents due to their unique physicochemical properties, including nanoscale dimensions, high surface-to-volume ratios, and surface tunability. These attributes impart enhanced antimicrobial efficacy, particularly against resistant pathogens, positioning NPs as promising tools in the ongoing fight against microbial infections [3].

NPs are broadly classified into inorganic and organic types, each exhibiting distinct characteristics, mechanisms of action, and synthetic routes designed for their specific applications [4]. Inorganic NPs, such as silver, gold, zinc oxide, and copper oxide, are widely recognized for their potent antimicrobial activity and stability under extreme conditions [5]. Their applications span diverse sectors, including medicine, agriculture, and environmental disinfection. However, their widespread use has raised significant concerns regarding their safe disposal, long-term fate, and potential environmental accumulation [6, 7]. These NPs can persist in ecosystems, potentially disrupting ecological balances and interacting with living organisms in ways that pose risks to human health and environmental sustainability [7]. They are nonbiodegradable, leading to their accumulation in the environment, particularly in aquatic and terrestrial ecosystems. This can disrupt ecosystems and harm plants, animals, and microorganisms [8]. Additionally, leaching toxic components such as heavy metals into soil and water can further contaminate the environment, creating severe risks. In humans, these materials are associated with cytotoxicity, inflammation, oxidative stress, and bioaccumulation, with long-term exposure potentially resulting in poorly understood health risks [7, 8]. Their limited biocompatibility, poor biodegradability, and potential to trigger unintended immune responses further constrain their biomedical applications [9].

In contrast, organic NPs represent a versatile and diverse group of nanomaterials composed of organic molecules such as polymers, lipids, and proteins. These can be further categorized into synthetic and biobased types. Synthetic organic NPs such as polymeric NPs, dendrimers, and liposomes are typically produced through controlled chemical processes using synthetic polymers and nonrenewable resources [10]. While these NPs benefit from well-established manufacturing techniques, defined composition, and tunable properties, they share environmental drawbacks similar to inorganic NPs, including potential toxicity, high energy consumption during production, and challenges related to waste disposal [11].

**Figure 1:**
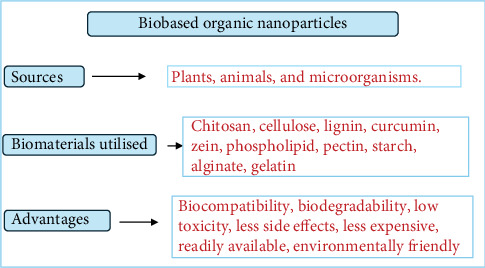
Sources, biomaterials utilised, and advantages of biobased organic NPs.

Biobased organic NPs, on the other hand, are synthesized from renewable, biodegradable sources such as plant extracts, bacteria, or fungi. As illustrated in Figure 1, these NPs offer notable environmental and biomedical advantages, including reduced toxicity, improved biocompatibility, and a lower carbon footprint [12].

In response to growing environmental concerns, green nanotechnology has emerged as a transformative approach that emphasizes the development of biobased organic NPs using ecofriendly synthesis routes and natural polymers such as chitosan, cellulose, lignin, and curcumin [13]. The use of ecofriendly NPs aligns with sustainable development goals, offering enhanced safety, reduced toxicity, and improved biocompatibility, making them ideal for antimicrobial applications. Their production from natural, renewable resources minimizes their environmental impact while retaining high therapeutic efficacy [14]. These NPs exhibit low cytotoxicity to human cells yet maintain potent antimicrobial activity [15, 16]. Their nanoscale size, high surface area, and functionalization potential enable effective interaction with microbial cells, disruption of biofilms, and broad-spectrum antimicrobial effects [3]. Moreover, biobased organic NPs can be engineered to target specific pathogens, thereby improving therapeutic precision and reducing off-target effects. The broad-spectrum antimicrobial activity of biobased organic NPs has been demonstrated against various pathogens, including gram-positive and gram-negative bacteria and fungi [17, 18]. These nanomaterials offer promising ways to combat AMR, addressing the limitations of conventional antibiotics by utilizing their unique physicochemical properties for targeted drug delivery and innovative therapeutic strategies [19].

This review provides an in-depth analysis of the potential of biobased organic NPs as antimicrobial agents, with a particular focus on their physicochemical properties and applications. Key factors influencing their antimicrobial efficacy, such as size, shape, surface charge, and stability, are examined. Furthermore, their advantages in drug delivery systems, including enhanced stability, controlled release, improved bioavailability, and targeted delivery, are also discussed. Despite their potential, challenges such as scalability, cost of production, and standardization of safety protocols remain. The review concludes by outlining future perspectives, offering strategies to optimize the development of biobased organic NPs. With continued innovation, biobased organic NPs hold great potential as sustainable solutions to urgent public health challenges.

## 2. Synthesis of Biobased Organic NPs

The synthesis of biobased organic NPs has gained significant attention due to the growing demand for sustainable, biodegradable, and nontoxic nanomaterials. Various green synthesis methods, such as ionic gelation, microemulsion, and mechanical techniques, have been developed to fabricate NPs from natural polymers and biomolecules [20]. These methods utilize mild conditions and environmentally friendly reagents to produce functional NPs tailored for applications in biomedicine, food, agriculture, and environmental remediation [10].

The ionic gelation method is a widely used approach for synthesizing biobased NPs from natural polymers like alginate, cellulose, and chitosan. This method relies on electrostatic interactions between oppositely charged components to form NPs. Alginate, a negatively charged polymer, cross-links with divalent cations such as calcium (Ca^2+^) [21], while chitosan, a positively charged polymer, interacts with polyanions like sodium tripolyphosphate (NaTPP) [22]. These interactions induce a sol-to-gel transition, resulting in the formation of stable NPs [23]. Van Bavel et al. [24] successfully produced monodisperse chitosan NPs with high yield, achieving a particle size range of 68–77 nm. This method operates under mild, aqueous, and solvent-free conditions.

The microemulsion method is another commonly used method for synthesizing stable NPs from biodegradable and organic polymers. Microemulsions are isotropic, thermodynamically stable, and transparent colloidal dispersions composed of at least three components: a polar phase, a nonpolar phase, and a surfactant, often forming either oil-in-water or water-in-oil systems [25]. The water-in-oil microemulsion approach is commonly used in NP synthesis, particularly with biodegradable polymers. This involves adding an aqueous polymer solution to an organic phase containing surfactants or cosurfactants, followed by homogenization to produce nanoscale droplets that serve as nanoreactors for polymerization or crosslinking reactions [20]. Once the emulsion is formed, NPs can be generated through several solidification techniques. Solvent-based precipitation is one of the most common, where biopolymers such as chitosan, gelatin, or alginate are precipitated as the solvent is gradually removed by evaporation, diffusion, or salting-out. Alternatively, gelation of emulsion droplets can be achieved through chemical or ionic cross-linking agents, leading to the formation of biocompatible NPs with stable, solid structures. A more advanced route involves in situ polymerization within the emulsion droplets. Monomers introduced into the system undergo polymerization through conventional, surfactant-free, or interfacial techniques allowing for precise control over particle size, architecture, and functional properties [26]. NPs are typically recovered through solvent evaporation or centrifugation [22]. Several studies have demonstrated the practical effectiveness of the microemulsion method. For instance, Duyen and Hung successfully fabricated starch NPs from debranched cassava starch. In their work, a starch solution was added to an ethanol-based organic phase containing Tween 80 and soybean oil and stirred at high speed for 1 h to form a microemulsion. The resulting NPs were collected through centrifugation and freeze-dried. Their average sizes varied with the starch's degree of polymerization (DPn): high DPn (> 35) produced 90.1 ± 0.3 nm particles, medium DPn (15–30) yielded 103.9 ± 0.4 nm, and low DPn (< 10) formed larger particles of 149.7 ± 0.5 nm, showing a clear link between polymer chain length and NP size [27]. In another study, Demirkurt et al. [28] developed bovine serum albumin (BSA) NPs using a novel water-in-ionic liquid microemulsion system. This system consisted of water as the dispersed phase, Tween 20 as the surfactant, and 1-butyl-3-methylimidazolium hexafluorophosphate, a hydrophobic ionic liquid, as the continuous phase. BSA solution at pH 9.0 was added dropwise to the mixture, followed by high-speed homogenization (22,000 rpm) and cross-linking with glutaraldehyde. The degree of cross-linking can be tailored to control drug release profiles. Substituting conventional solvents and surfactants with ionic liquids offers a green chemistry alternative, enhancing sustainability by reducing toxicity [29].

The nanoprecipitation method, also known as solvent–antisolvent precipitation, is another simple, cost-effective technique for synthesizing biobased organic NPs. It involves two miscible phases, an organic phase (typically a polymer or bioactive compound dissolved in a good solvent) and an aqueous phase (a poor solvent or antisolvent). When the organic phase is added to the aqueous phase under constant agitation, the difference in polarity and surface tension between the solvents, along with their miscibility, induces rapid supersaturation, leading to the formation of a colloidal NP suspension [30]. For example, curcumin NPs were synthesized from *Curcuma xanthorrhiza* Roxb. extract by gradually adding ethanol-extracted curcumin into distilled water under continuous stirring, resulting in NPs with an average size of 164.37 ± 3.29 nm [31].

Mechanical methods utilize physical forces to reduce biobased organic materials to the nanoscale without altering their chemical structure. These techniques are important to green nanotechnology, as they eliminate the use of hazardous chemicals and can be applied to diverse natural materials such as plant extracts, polysaccharides, and proteins [32]. Among these, ball milling is a widely employed technique wherein grinding balls in a rotating chamber break down materials like cellulose, starch, and lignin into NPs. For example, Verma et al. [33] prepared bamboo nanocellulose by subjecting bamboo fibers to sequential alkaline and acid treatments to remove noncellulosic components, followed by ball milling at 500 rpm for 4 h, reducing particle size from ~500 *μ*m to ~80 nm. Similarly, Gnanasekaran et al. [34] synthesized nanofibrillated cellulose (NFC) from *Ananas comosus* leaf fiber through steam-alkaline and bleaching pretreatments, followed by ball milling with 1 mm zirconia balls at 700 rpm, yielding NFC with a diameter of 25.84 ± 8.30 nm. High-pressure homogenization is another effective method, in which a fluid containing biomaterials is forced through a narrow orifice under high pressure, generating intense shear and cavitation forces that reduce particle size. Samsalee et al. [35] applied high-pressure homogenization to produce cellulose nanofibers (CNFs) from rice husk. After alkaline and peroxide bleaching treatments to remove lignin, hemicellulose, and silica, the cellulose was homogenized at 120 MPa for multiple cycles, yielding CNFs with diameters of 10.15–12.42 nm and lengths of 602.13–620.08 nm. Ultrasonication or ultrasound-assisted synthesis uses high-frequency sound waves to generate acoustic cavitation in liquids. This process fragments suspended materials and is particularly useful for NP suspensions derived from organic extracts or proteins. For instance, Long et al. [36] produced garlic oil nanoemulsions by emulsifying garlic oil with Tween 80 and Span 80 in water and then ultrasonicated the mixture at 80% amplitude for 3–5 min using a JY92-IIN Scientz processor, yielding droplets with a mean size of 52.27 nm. Similarly, Anjum et al. [37] formulated water-in-oil nanoemulsions with *Tinospora cordifolia* extract using ultrasonication, achieving particle sizes ranging from 171.90 to 293.66 nm. Additionally, plant extracts have been nanosized in their pure form using this method, without chemical surfactants or stabilizers, offering a safer, greener alternative to traditional methods where plant extracts served as capping or reducing agents for metallic NP synthesis. For instance, Moringa leaf NPs were synthesized by extracting 400 mg of dried leaf powder in methanol and spraying it into boiling water under ultrasonic conditions, resulting in spherical NPs averaging 141.6 nm [38]. Similarly, curcumin NPs were synthesized from raw turmeric rhizomes through Soxhlet extraction, followed by dropwise addition of the extract to boiling water under ultrasonication, producing spherical NPs averaging 82 ± 4 nm [39]. Other mechanical methods like microfluidization and cryomilling also support ecofriendly NP production [40, 41].

The feasibility of producing biobased organic NPs is highly promising in sustainable and green nanotechnology. Derived from renewable materials like plant extracts, polysaccharides, and proteins, these NPs offer an ecofriendly alternative to synthetic ones, supporting circular economy principles. Their biodegradability, biocompatibility, and bioactivity make them ideal for diverse applications in healthcare and medicine, food packaging, cosmetics, and environmental remediation [42].

However, several challenges remain for large-scale production. Raw material variability, environmental sensitivity, and achieving uniform particle size are major issues. Energy-intensive methods like ball milling and high-pressure homogenization also pose scalability and cost challenges [12, 43]. Additionally, low solubility and bioavailability of certain compounds (e.g., curcumin) complicate formulation efforts, particularly without stabilizers.

While materials like starch and cellulose have been commercialized due to their cost-effectiveness, others like lignin, chitosan, and alginate face technical hurdles related to extraction processes and scalability [43]. Overcoming these challenges will require refining extraction techniques and improving green synthesis methods that minimize chemical use and reduce energy consumption.

Advancing green synthesis and improving NP performance and stability are key to unlocking their full potential. With continued research, innovation, and collaboration, biobased NPs can become a cornerstone of sustainable nanotechnology, expanding applications and contributing to a circular economy.

## 3. Mechanisms of Antibacterial Action of Biobased Organic NPs

Biobased organic NPs have emerged as promising antimicrobial agents due to their diverse mechanisms of action, ecofriendly nature, and potential to combat antibiotic-resistant pathogens. These NPs exhibit a multifaceted approach to bacterial inhibition, targeting structural, metabolic, and regulatory pathways within microbial cells.

### 3.1. Disruption of Bacterial Cell Walls and Membranes

Biobased organic NPs disrupt bacterial cell walls through direct interaction, causing mechanical damage and increasing membrane permeability. Additionally, positively charged NPs attract and interact with the negatively charged bacterial membranes, destabilizing their structure and leading to the leakage of intracellular contents [44, 45]. For example, Gao et al. [46] demonstrated that lignin NPs disrupt bacterial cell walls and membranes with species-specific effects. *Escherichia coli* showed rapid damage within 2 h, while *Staphylococcus aureus* exhibited severe destruction by 4 h. Scanning electron microscopy (SEM) revealed deformed cells and intracellular leakage after 12 h. Increased conductivity in *E. coli* cultures indicated altered membrane permeability, highlighting lignin NPs' role in compromising bacterial integrity and promoting cell death. Xing et al. [47] also demonstrated that chitosan NPs exert antifungal activity against *Paecilomyces steckii and Aspergillus oryzae* by disrupting fungal cell membranes, causing cytoplasmic leakage, and ultimately resulting in cell death.

### 3.2. Induction of Oxidative Stress Through Reactive Oxygen Species (ROS) Generation

Biobased NPs exert antimicrobial effects by generating ROS, which induce oxidative stress in bacteria. This process damages cellular components, including DNA, lipids, and proteins, ultimately disrupting metabolism and leading to cell death. Morena et al. [48] demonstrated that lignin-based NPs trigger ROS production, significantly reducing bacterial metabolic activity and compromising cell viability. Similarly, curcumin NPs enhance antibacterial activity under light exposure by generating ROS, which disrupts bacterial membranes and DNA, inhibiting growth [49, 50]. Work by Roquito et al. [51] showed that curcumin-loaded glucan NPs have demonstrated a significant ability to induce oxidative stress in cells. This effect is mediated by the stimulation of ROS, nitric oxide (NO), and the chemokine RANTES, with their levels increasing in a concentration-dependent manner. Although these findings were observed in cancer cells rather than bacterial cells, they underscore the potential of curcumin-loaded glucan NPs as a promising therapeutic strategy, leveraging oxidative stress to effectively target and disrupt cell viability [51].

### 3.3. Biofilm Disruption and Quorum Sensing (QS) Inhibition

NPs can disrupt the extracellular polymeric substances of biofilms. By targeting the structural integrity of biofilms, NPs increase bacterial susceptibility to antimicrobial treatments, overcoming one of the key defenses used by pathogenic bacteria [52]. Additionally, NPs interfere with QS, a vital bacterial communication system that governs biofilm formation and the expression of virulence factors. QS is crucial for pathogens to coordinate activities necessary for survival, colonization, and infection. Interrupting QS pathways offers a promising, nonlethal strategy to reduce bacterial pathogenicity without directly inducing selective pressure that may lead to resistance [53]. A study by Fattah et al. [54] highlighted the potential of chitosan NPs to inhibit QS-regulated virulence factors in *Pseudomonas aeruginosa*. The study demonstrated that chitosan NPs effectively suppressed motility, biofilm formation, and the expression of key QS-related genes, *LasI* and *RhlI*. These findings emphasize the potential of biobased NPs as an innovative approach for managing bacterial infections and combating AMR [54].

### 3.4. Intracellular Interference With Genetic and Metabolic Processes

Biobased organic NPs penetrate microbial cells and disrupt essential intracellular processes [55]. They interact with microbial DNA, inhibiting replication and transcription, while also interfering with key enzymes vital for metabolism and survival [56]. Liu et al. [57] demonstrated that chitosan strongly binds to DNA, likely due to interactions between its amino groups and the negatively charged phosphate groups on DNA. This interaction enables NPs to effectively prevent bacterial proliferation. For instance, oleoyl-chitosan NPs have been reported to exert antibacterial effects by internalizing into the cell, where they bind to DNA and RNA [58]. Rai et al. [59] demonstrated that curcumin disrupts bacterial cell division by targeting FtsZ, a key protein in cytokinesis. Their study revealed that curcumin binds to FtsZ, enhancing its GTPase activity and preventing Z-ring formation. This disruption inhibits cell division, ultimately leading to bacterial growth suppression and cell death [59].

Chitosan and its derivatives exhibit antifungal activity by targeting intracellular processes in fungi. In *Candida albicans*, chitosan represses the *MSS2* gene, disrupting mitochondrial function and ATP production, weakening virulence. This disruption compromises the pathogen's ability to thrive and cause infection, highlighting chitosan's potential as an effective antifungal agent [60]. Xia et al. [61] showed that hydroxypropyltrimethyl ammonium chloride chitosan causes mitochondrial transmembrane potential collapse, increased membrane swelling, and reduced membrane fluidity, indicating the opening of the mitochondrial permeability transition pore.

### 3.5. Inhibition of Nutrient Uptake and Metal Ion Deprivation

Biobased organic NPs exhibit antimicrobial activity by depriving bacteria of essential metal ions required for metabolism, enzyme function, and structural stability. Chitosan, for example, acts as a chelating agent, binding Ni^2+^, Zn^2+^, Co^2+^, Fe^2+^, and Cu^2+^, especially at pH levels above its pKa [62]. Chelating agents have molecular structures with multiple binding sites that form stable complexes with metal ions, effectively sequestering them and preventing bacterial access to vital nutrients like iron, zinc, and manganese. This disruption in metal homeostasis leads to metabolic stress, impaired enzyme function, and weakened cell wall integrity, ultimately inhibiting bacterial growth [63]. This mechanism highlights the potential of biobased NPs as effective antimicrobial agents.

Biobased organic NPs provide a sustainable and ecofriendly alternative for combating bacterial infections, particularly against antibiotic-resistant pathogens. Their multifaceted mechanisms make them promising candidates for medical, agricultural, and industrial applications.

## 4. Factors that Influence the Antimicrobial Activity of Naturally Derived Organic NPs

**Figure 2:**
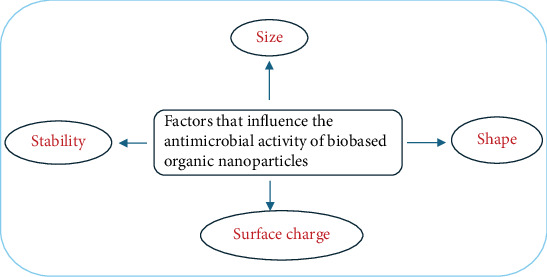
Factors influencing the antimicrobial activity of biobased organic NPs.

Naturally derived organic NPs have emerged as promising antimicrobial agents due to their biocompatibility, ecofriendliness, and ability to combat a wide spectrum of microbial infections, including drug-resistant strains [64]. Their antimicrobial efficacy is influenced by various physicochemical properties, including particle size, shape, surface charge, and stability (Figure 2), as well as their interactions with microbial targets. These properties determine the NPs' ability to penetrate biofilms, interact with microbial membranes, and disrupt cellular processes [3].

Table 1 presents a detailed summary of naturally derived organic NPs, highlighting their characteristics, stability, and antimicrobial properties. This compilation indicates the diversity of these NPs in terms of structural attributes and their varied effects against different pathogens. Factors such as size-dependent interactions, surface charge-mediated attraction or repulsion, and environmental stability play crucial roles in modulating their antimicrobial activity. Understanding these factors is essential for optimizing the design and application of organic NPs in antimicrobial strategies.

### 4.1. NP Size

NP size is a crucial factor in determining their antibacterial efficacy, as it directly affects interactions with microbial cells. Smaller NPs generally exhibit enhanced activity due to their high surface area-to-volume ratio, which enhances contact with bacterial membranes and facilitates biological processes such as endocytosis, distribution, and cellular retention [83, 84]. This property enables materials that are inert in bulk form to become reactive at the nanoscale. For example, curcumin NPs (87 ± 8 nm) exhibited significantly greater antibacterial activity than bulk curcumin, producing larger inhibition zones against *S. aureus* (29.91 ± 0.53 mm) and *E. coli* (24.58 ± 1.12 mm) compared to their bulk counterpart (with inhibition zones of 24.82 ± 0.54 mm for *S. aureus* and 19.70 ± 1.18 mm for *E. coli*) [85]. Similarly, lignin (Lig) NPs (50–350 nm) outperformed bulk lignin, showing enhanced antioxidant properties, improved protein adsorption, superior bactericidal activity, and accelerated wound healing in in vivo mouse studies [86]. Reduction of herbal extracts like propolis and cinnamon to the nanometer scale also increased their antibacterial activity against *S. mutans* and their antioxidant potential [87].

Smaller NPs consistently show greater activity than larger ones. For instance, carbon dots (CDs) of ~2.0 nm demonstrated higher antimicrobial activity against *E. coli* and *S. aureus* than larger CDs (~3.9 and ~5.3 nm), attributed to improved cellular uptake [88]. Similarly, 40 nm NPs disrupted bacterial (*B. subtilis*, *S. epidermidis*, *E. coli*, and *P. aeruginosa*) membranes more effectively than their 58 nm counterparts [89]. These enhanced properties result from the increased surface area and better penetration of smaller NPs, enabling them to disrupt cellular processes more efficiently [53, 90].

In addition to direct antimicrobial action, NP size significantly affects loading capacity for bioactive compounds. For instance, reducing the size of periodic mesoporous organosilica NPs drastically increased their curcumin loading capacity, achieving 1984 mg of curcumin per gram of NPs [91]. This enhancement is particularly valuable for compounds like curcumin, which face challenges related to poor solubility and bioavailability [92]. Optimizing NP size not only improves therapeutic efficacy but also expands their potential in drug delivery and antimicrobial applications [93, 94].

As shown in Table 1, naturally derived organic NPs of various sizes have demonstrated antimicrobial activity against a wide range of microorganisms. For instance, chitosan NPs (28.5–364.4 nm) effectively inhibited biofilm formation and exhibited broad-spectrum activity against *P. aeruginosa*, *M. tuberculosis*, *E. coli*, *S. aureus*, *E. faecalis*, and *C. albicans* [17, 65, 67]. Similarly, curcumin NPs, ranging from 9 to 98.7 nm, displayed potent antibacterial activity against methicillin-resistant *S. aureus* (MRSA), *B. cereus*, *S. typhi*, *E. coli*, and antifungal activity against *C. glabrata* and *A. niger* [76–81]. Lignin NPs (40–350 nm) have also shown significant antimicrobial efficacy against *S. aureus*, *P. syringae*, *E. coli*, *B. cereus*, and other pathogens, with improved performance attributed to their nanoscale size and functional surface properties [18, 46, 48, 73, 74]. Furthermore, cellulose nanocrystals (CNCs) (~150 nm) inhibited bacterial growth, induced aggregation, and prevented biofilm formation in *P. syringae* [69, 70]. These findings highlight the critical role of small particle size in determining their activity and interaction with microbial targets.

### 4.2. NP Shape

The shape of NPs plays a critical role in determining their antimicrobial properties [95, 96]. While spherical NPs are extensively studied and consistently exhibit strong antimicrobial activity due to their uniform surface, anisotropic shapes such as nanorods, nanostars, and nanocubes have garnered attention for their enhanced antimicrobial potential. Research by Ray et al. [97] and Agrawal et al. [98] highlights that anisotropic forms like nanostars, nanorods, and nanocubes exhibit stronger antibacterial effects than spherical NPs. The elongated or sharp geometries of these shapes allow deeper microbial cell penetration and increased physical interaction, resulting in more effective cellular disruption and interaction with intracellular components [99, 100].

Despite the potential of anisotropic shapes, most research on shape-dependent antimicrobial effects has centered on inorganic NPs. For biobased organic NPs, spherical shapes dominate the literature [66], likely due to their simpler and more stable synthesis using conventional techniques like solvent evaporation and emulsion methods [101].

As shown in Table 1, naturally derived organic NPs with various shapes, including spherical, nearly spherical, cylindrical, rod-like, and acicular forms, have demonstrated antimicrobial activity against a broad range of microorganisms [46, 66, 67]. For example, spherical curcumin NPs have shown potent antibacterial properties against *S. aureus*, *E. coli*, and *B. cereus* [14]. Similarly, nearly spherical chitosan NPs have been effective against pathogens such as *P. aeruginosa* and *H. pylori* [65, 66]. Cylindrical and rod-like lignin NPs have also shown significant activity, particularly in reducing infections caused by *P. syringae* and other plant pathogens [69, 70]. However, spherical and nearly spherical NPs remain the most widely reported for antimicrobial applications, likely due to their easier synthesis compared to anisotropic counterparts. Further research into the development and synthesis of biobased anisotropic NPs could provide valuable insights into the role of shape in their antimicrobial efficacy, enhancing their effectiveness and paving the way for more sustainable therapeutic solutions.

### 4.3. NP Surface Charge

The surface charge of NPs plays a crucial role in their antimicrobial activity. Positively charged NPs are particularly effective as they attract negatively charged microbial cell membranes, facilitating binding, penetration, and disruption of cellular functions [90, 102]. Gram-positive bacteria have a cell wall composed of peptidoglycan and lipoteichoic acid, while Gram-negative bacteria possess an outer membrane rich in lipopolysaccharides, both contributing to a negative surface charge. This charge interaction enhances the binding of positively charged NPs, boosting antibacterial efficacy [3]. For example, bacteria such as *K. pneumoniae*, *E. coli*, *S. aureus*, and *P. aeruginosa* have been shown to exhibit high sensitivity to chitosan NPs, attributed to the cationic amine groups in chitosan that strongly bind to their negatively charged cell surfaces [103]. TEM micrographs have further demonstrated that chitosan NPs attach to the membranes of pathogens such as *P. fluorescens*, *Aeromonas hydrophila*, *Yersinia ruckeri*, *P. putida*, *A. caviae*, and *A. veronii*, disrupting their structural integrity [104].

On the other hand, a negative charge on NPs can reduce antimicrobial effectiveness by causing repulsion between similarly charged particles and microbial membranes, thereby hindering their interaction [105]. In some instances, negatively charged NPs can exhibit significant antimicrobial activity, which is influenced by microbial cell wall composition, membrane properties, and additional interactions beyond electrostatic forces, such as hydrophobic interactions, van der Waals forces, and hydrogen bonding. For example, unmodified lignin NPs, which naturally possess a negative surface charge due to the abundance of phenolic and carboxylic acid groups, have shown remarkable broad-spectrum antimicrobial properties. As shown in Table 1, LigNPs have demonstrated efficacy against a diverse array of pathogens, including bacteria and fungi [18, 48, 73], likely attributed to the antibacterial activity of functional groups such as phenolic groups [106]. This highlights the versatility of LigNPs as a sustainable and effective antimicrobial agent, despite their negative charge.

Interestingly, negatively charged NPs are utilized in specific applications to target positively charged groups. For example, Marques et al. [107] demonstrated that incorporating polyphosphate into chitosan produced negatively charged NPs (100–200 nm) capable of effectively encapsulating positively charged proteins. This finding highlights the versatility of negatively charged NPs, indicating their potential to be customized for targeting specific positively charged cellular components in microorganisms. Neutral-charge NPs are generally less effective as antimicrobials, as they lack strong electrostatic interactions with microbial membranes. This reduces their ability to bind and disrupt microbial cells effectively.

The surface charge of NPs can be controlled through various methods. Adjusting the pH of the synthesis medium allows for charge manipulation, with lower pH levels typically increasing positive charge and higher pH levels enhancing negative charge [108]. The use of surfactants as stabilizing agents can also modulate surface charge, as cationic surfactants increase positive charge, while anionic surfactants enhance negative charge. For instance, Richter et al. [109] utilized a cationic polyelectrolyte, polydiallyldimethylammonium chloride, to control the surface charge of lignin NPs. Functionalizing NPs with charged groups, such as carboxyl or amino groups, further enables tailored surface charge for improved interactions with target cells.

### 4.4. NP Stability

The stability of NPs is critical for maintaining their antimicrobial effectiveness. Stable NPs retain their size, shape, and surface properties over time, ensuring consistent and prolonged activity. In contrast, unstable NPs may aggregate or degrade, leading to reduced efficacy by altering their size, shape, or surface charge [110], which impairs their ability to interact with and disrupt microbial cells.

Ensuring NP stability during synthesis is vital for optimizing their performance in antimicrobial applications. For instance, Maršík et al. [65] addressed the challenge of NP instability by incorporating quaternized chitosan, which enhanced colloidal stability in culture media. This approach resulted in trimethylchitosan NPs with a stability of up to 1 month in aqueous stock solutions [65]. Such advancements demonstrate the necessity of stabilizing strategies to preserve biological activity.

Several strategies can address stability challenges. Incorporating stabilizing agents, such as cationic, anionic, or nonionic surfactants, prevents aggregation by sterically hindering particle interactions. Adjusting the pH of the synthesis medium enhances the ionization of surface functional groups, improving electrostatic repulsion and colloidal stability [52]. Olivas-Flores et al. [17] demonstrated the critical role of pH in determining the stability and size of chitosan NPs. At pH 4, chitosan NPs exhibited a positive zeta potential of 40 ± 3 mV, a result of the protonation of amino and hydroxyl groups under acidic conditions. This protonation promoted the formation of hydrogen bonds, enhancing NP stability. Smaller chitosan NPs were particularly stable, maintaining sizes of 87.3 ± 1.1 nm and 146.0 ± 8.0 nm even under prolonged storage. However, larger NPs exhibited a notable size increase over time, reaching 219.2 ± 10.1 nm and 274.7 ± 20.7 nm after 1 month of refrigerated storage at 4°C–8°C. When the pH increased, the size of the NPs expanded dramatically, eventually reaching micrometer dimensions [17]. This phenomenon was attributed to the neutralization of chitosan's positive charges upon the addition of NaOH. The resulting reduction in electrostatic repulsion allowed the NPs to aggregate, forming larger clusters.

Controlling ionic strength is also vital, as excessive salts can shield surface charges and promote aggregation, while optimal levels preserve stability [17, 111]. Additional strategies include temperature regulation, surface functionalization, and reaction time optimization, all of which prevent aggregation and ensure consistency. Morsali et al. [112] demonstrated that the instability of lignin NPs can be resolved by synthesizing hydroxymethylated lignin NPs followed by hydrothermal curing, which stabilizes the particles through internal cross-linking reactions. The resulting colloidally stable NPs exhibit a high biobased content of 97%, with a tunable particle size distribution and robust structural stability in aqueous media across a broad pH range (3–2) and in organic solvents such as acetone, ethanol, dimethylformamide, and tetrahydrofuran [112].

The zeta potential is a reliable indicator of NP stability, with values exceeding +30 mV or below −30 mV typically signifying good stability [113]. Yang et al. [74] confirmed this, reporting zeta potential values lower than −30 mV for LigNPs, attributed to their high content of phenolic moieties. Moreover, LigNPs demonstrated exceptional stability, remaining stable for over a year in aqueous conditions at 5°C without any observable precipitation [74]. This stability is due to electrostatic repulsions between the negatively charged particles, resulting in electrosteric stabilization. These findings highlight the significance of employing tailored strategies to ensure NP stability, particularly in diverse applications such as antimicrobial treatments.

Understanding factors influencing the antimicrobial activity of naturally derived organic NPs is pivotal for optimizing their design and application in combating microbial infections.

## 5. Applications Biobased Organic NPs in Disease Management

Biobased organic NPs have demonstrated significant potential in disease management. This section highlights two key applications of biobased organic NPs in the field of disease management:

### 5.1. Antimicrobial Properties Against Pathogens

Biobased organic NPs such as chitosan, cellulose, lignin, and curcumin (as shown in Table 1) have demonstrated potent antimicrobial properties against a variety of pathogens, including bacteria and fungi. These properties make them invaluable tools for controlling infectious diseases.

#### 5.1.1. Antimicrobial Properties of Chitosan NPs

Chitosan, derived from chitin found commonly in the exoskeletons of arthropods and crustaceans like shrimp, crab, and lobster, is produced by deacetylating chitin. This biopolymer exhibits strong antimicrobial properties, is highly biocompatible, biodegrades naturally without harmful residues, and is nontoxic [114, 115]. These attributes make chitosan a valuable antimicrobial agent in the food and pharmaceutical industries. The development of chitosan NPs has further enhanced these properties, improving antimicrobial efficacy, controlled release, and stability [114].

As illustrated in Table 1, chitosan NPs exhibit broad-spectrum activity against both bacterial and fungal pathogens [3, 17, 65–67]. Their antimicrobial action involves multiple mechanisms, primarily electrostatic interactions between the positively charged chitosan NPs and the negatively charged anionic surfaces of Gram-positive and Gram-negative bacteria. These interactions destabilize bacterial membranes, causing structural disruption, leakage of intracellular components like nucleotides and proteins, and penetration of chitosan NPs into the cytoplasm, where they disrupt internal processes [116].

In fungi, chitosan NPs interact with phosphorylated mannosyl residues on cell walls, leading to plasma membrane disruption and leakage of intracellular materials, which compromises fungal viability [117, 118]. Additionally, chitosan NPs chelate metal ions on microbial surfaces through their amino groups. When the chelation effect outweighs electrostatic forces, it further destabilizes the microbial cell membrane [116, 119]. These mechanisms show the potential of chitosan NPs as effective antimicrobial agents, particularly valuable in combating microbial infections.

#### 5.1.2. Antimicrobial Properties of CNCs

Cellulose, a natural polymer found in plant cell walls and the most abundant organic polymer, is sourced from materials such as wood, cotton, agricultural residues, grasses, and algae [120, 121]. Through nanosizing, cellulose can be transformed into CNCs or CNFs. This transformation enhances its properties, such as increased surface area, improved mechanical strength, and unique optical features. Nanosized cellulose exhibits superior interactions with other materials, making it valuable in various applications including biocomposites [122], drug delivery systems [123, 124], and antimicrobial agents [70]. Additionally, nanocellulose is nontoxic, biodegradable, and biocompatible, posing no adverse effects on health or the environment.

As shown in Table 1, CNCs exhibit significant antimicrobial activity against a range of bacterial pathogens, including *P. syringae*, *E. coli*, and *P. savastanoi*. Their antimicrobial mechanism involves multiple strategies, such as direct bacterial inactivation, disrupting community structures like biofilms, inducing bacterial aggregation, and potentially enhancing host immune defenses [69–72]. Noronha et al. [71] demonstrated that CNCs inhibited approximately 90% of *E. coli* cells. This was attributed to the disruption of membrane integrity, a mechanism supported by evidence from SEM and dye-encapsulated phospholipid vesicle leakage assays. These analyses revealed that CNCs physically damage bacterial cell membranes, leading to cell death [71].

This multifaceted and broad-spectrum activity underscores the potential of CNCs as effective agents for antimicrobial applications, particularly in areas requiring sustainable and versatile solutions, such as healthcare, agriculture, and food preservation.

#### 5.1.3. Antimicrobial Properties of Lignin NPs

Lignin, a complex polymer providing structural support to plants, is mainly sourced from wood, agricultural residues, and grasses, with significant amounts also produced as a by-product of the pulp and paper industry [125]. Nanosizing lignin enhances its properties by increasing surface area, improving dispersibility, and boosting reactivity [126]. These nanosized lignin particles exhibit superior antioxidant, antimicrobial, and UV-blocking properties, making them highly valuable in applications such as biocomposites, drug delivery systems, molecule encapsulation, and packaging materials [127]. LigNPs are particularly attractive due to their biodegradability, biocompatibility, cost-effectiveness, and nontoxicity, promoting their use in health and environmental applications.

As displayed in Table 1, LigNPs exhibit strong antibacterial activity against a wide range of bacterial and fungal pathogens, with their effectiveness significantly enhanced by chemical modifications [18, 48, 73]. Functionalization strategies, such as incorporating tannic acid, increase the phenolic content of LigNPs. This leads to the development of phenolic acid-functionalized LigNPs (PheLigNPs), which demonstrate enhanced antibacterial activity compared to nonfunctionalized LigNPs and bulk phenolated lignin. These improvements are attributed to the synergistic effects of elevated phenolic content and the nanoscale size of the particles [48].

Additionally, Ali et al. [18] established that LigNPs show greater efficacy against bacterial pathogens than fungal ones. The mechanism of action involves disrupting microbial cell integrity and interfering with protein synthesis, thereby impairing critical cellular functions [18]. The dual capability of LigNPs to act on different types of pathogens, along with their modifiable nature, makes them strong candidates for innovative antimicrobial agents in fields such as medicine, agriculture, and food safety.

#### 5.1.4. Antimicrobial Properties of Curcumin NPs

Curcumin, a natural polyphenol from the turmeric plant (*Curcuma longa*), is renowned for its strong antioxidant, anti-inflammatory, and antimicrobial properties [128]. While it shows promise in managing inflammation, oxidative stress, microbial infections, and cancer treatment, its effectiveness is hindered by poor water solubility and low gastrointestinal absorption [129, 130]. Nanosizing curcumin addresses these challenges, improving its bioavailability and enhancing drug delivery for better therapeutic outcomes [131]. As shown in Table 1, curcumin NPs exhibit strong antibacterial activity against a diverse range of pathogens, including various bacteria and fungi. The NPs display significantly improved antimicrobial effectiveness compared to bulk curcumin [76–79]. This enhanced efficacy is primarily due to their increased solubility, smaller particle size, and optimized surface properties, which facilitate better interaction with microbial cells [81].

Curcumin NPs combat bacteria through multiple mechanisms, including damaging bacterial membranes, generating ROS that harm vital cell components, disrupting DNA replication and protein synthesis, inhibiting QS and biofilm formation, and interfering with cell wall synthesis. These actions collectively impair bacterial growth, structure, and defenses, making Curcumin NPs effective antibacterial agents [132, 133].

#### 5.1.5. Antimicrobial Properties of Biobased Organic NP Composites

Composites have shown remarkable potential as antimicrobial agents due to their synergistic properties. For example, curcumin and pectin NP composites, as well as nisin-pectin NP composites (Table 1), have demonstrated substantial antibacterial activity against significant pathogens like *S. aureus* and *E. coli* [82]. These composites work through multiple mechanisms, such as disrupting bacterial membranes, inhibiting biofilm formation, and interfering with bacterial metabolism. Their enhanced efficacy is attributed to the combination of antimicrobial agents, which improve stability, bioavailability, and target specificity.

The application potential of these composites spans various fields. In medicine, they could serve as advanced wound dressings or drug delivery systems to combat infections. In food preservation, they act as active packaging materials that inhibit spoilage and extend shelf life by controlling microbial growth. Similarly, in the packaging industry, they provide a biodegradable and antimicrobial alternative, reducing reliance on conventional plastics while ensuring product safety. Their versatility and robust bacterial control highlight their promise as innovative solutions for addressing challenges in microbial contamination and infection control.

### 5.2. Biobased Organic NPs for Drug Delivery

Biobased organic NPs have gained significant attention as effective drug delivery systems, particularly in antimicrobial applications [134]. These NPs are derived from natural polymers like chitosan, cellulose, starch, and lignin, which provide biocompatibility, biodegradability, and enhanced drug encapsulation efficiency [135, 136]. Their ability to encapsulate antimicrobial agents provides a promising approach for improving the efficacy and targeting of drugs against various bacterial pathogens.

Table 2 below summarizes recent studies on biobased organic NPs used as drug carriers, demonstrating their potent antibacterial activity. The table highlights the effects of different formulations against a range of pathogens, such as *P. aeruginosa*, *E. coli*, and *S. aureus*, along with the effects of their action.

The encapsulation of drugs or bioactive compounds within or onto biobased NPs, as detailed in Table 2, provides several advantages.

#### 5.2.1. Enhanced Stability and Protection

Encapsulation improves the stability of bioactive compounds by protecting them from environmental degradation caused by factors like light, oxygen, pH fluctuations, temperature changes, and other harsh conditions of the gastrointestinal tract [162]. For instance, tea polyphenols, epigallocatechin gallate, and curcumin, which are prone to oxidation and degradation, retain their antioxidant and therapeutic properties when encapsulated [163]. This protection is crucial for maintaining their efficacy during storage and application. Similarly, essential oils like *Z. officinale*, *C. cyminum*, and *Z. multiflora*, which are volatile and susceptible to degradation [164], benefit from encapsulation as it reduces their volatility, thus preserving their antimicrobial and therapeutic effects. In both cases, encapsulation not only prolongs the shelf life of these compounds but also maintains their antibacterial efficacy.

#### 5.2.2. Controlled and Sustained Release

Encapsulation enables controlled and sustained release of active compounds, enhancing therapeutic outcomes while minimizing side effects [165]. For instance, carvacrol and carvacrol@casein NPs exhibit gradual release, effectively enhancing antimicrobial activity against pathogens like *E. coli*, *S. aureus*, and *B. cereus* over time, aiding in efficient infection control with minimal resistance development [140, 145]. Similarly, encapsulating antibiotics such as ceftazidime, ciprofloxacin, rifaximin, and ceftizoxime in biobased organic NPs (Table 2) enables controlled release, reducing dosing frequency [137, 144]. For example, Campos et al. [137] demonstrated that chitosan-coated zein NPs provide an effective delivery system for ceftazidime and tobramycin against antibiotic-resistant and biofilm-producing pathogens like *P. aeruginosa* and *K. pneumoniae*. The release kinetics showed a two-phase profile: an initial rapid release followed by sustained release for up to 24 h in simulated gastrointestinal conditions [137]. The chitosan coating forms a dense barrier around the NPs, regulating antibiotic release and enhancing drug efficacy and bioavailability while minimizing side effects and dosing frequency [166]. This sustained release ensures therapeutic drug levels are maintained over extended periods, improving treatment efficacy and minimizing toxicity risks. By providing consistent drug activity, encapsulation significantly improves the safety and effectiveness of active compounds, making them ideal for long-term therapeutic applications.

#### 5.2.3. Improved Bioavailability

Encapsulation significantly enhances the bioavailability of bioactive compounds, especially those with poor solubility, which often limits their therapeutic potential. By improving absorption and efficacy, encapsulation enables these compounds to reach their target sites more effectively [167]. For instance, curcumin, known for its low water solubility, benefits greatly from encapsulation, which improves its bioavailability and broad-spectrum antimicrobial activity. As highlighted in Table 2, curcumin-loaded starch–based nanocapsules demonstrate potent antibacterial effects against *E. coli* and *S. aureus* [149]. Similarly, curcumin encapsulated in LigNPs exhibits significant activity against a wider range of pathogens, including *S. aureus*, *E. coli*, and *P. aeruginosa* [153]. Encapsulation within LigNPs not only enhances curcumin's bioavailability but also provides a sustained release mechanism, ensuring consistent antimicrobial action over time. Trans-resveratrol and rutin face similar challenges due to low solubility and limited bioavailability, which restrict their therapeutic applications [168, 169]. Encapsulation into chitosan NPs improves their stability and absorption, ensuring efficient delivery to target tissues, as shown by their antimicrobial activity against *P. aeruginosa* and *H. pylori* [139, 142]. Furthermore, chitosan NPs enable controlled and sustained release of the encapsulated agents, enhancing therapeutic efficacy over time while reducing potential side effects. Encapsulation thus serves as a vital strategy to address the limitations of poor solubility, unlocking the full therapeutic potential of these bioactive compounds.

#### 5.2.4. Targeted Delivery

Targeted delivery is a key advantage of encapsulating bioactive compounds, as it allows for the precise delivery of drugs to specific sites of infection or disease, minimizing systemic side effects [170]. This approach improves therapeutic efficacy by delivering active compounds to the target site at higher concentrations while minimizing exposure to healthy tissues [171]. For example, encapsulating MRSA phage [141] and LysMR-5 [160] to chitosan-based NPs (as indicated in Table 2) enables these therapeutic agents to be specifically targeted to bacterial infections, effectively combating the resistant pathogens while minimizing the risk of off-target effects. Similarly, encapsulating nisin, an antimicrobial peptide, in NPs enhances its stability and improves its effectiveness in targeting microbial biofilms [124, 155]. Biofilms, which often protect bacteria from conventional antibiotics, can be difficult to treat, but with encapsulation, nisin can be delivered more efficiently to the biofilm's core, increasing its antimicrobial activity [124, 172]. In both cases, encapsulation ensures that the drugs are delivered where they are most needed, improving therapeutic outcomes and minimizing the risk of unwanted side effects, which is especially important for targeted treatments of localized infections.

#### 5.2.5. Enhanced Antimicrobial Activity

Enhanced antimicrobial activity is one of the key benefits of encapsulating antimicrobial agents [173, 174], as it improves their effectiveness by increasing penetration into biofilms or extending their activity over time. Biofilms, which are protective layers formed by microbial communities, often reduce the effectiveness of conventional treatments [173]. Encapsulation allows antimicrobial agents to better penetrate these biofilms, ensuring that they reach the deeper layers of the microbial community where they are most needed. For example, encapsulating chitosan and gallic acid enhances their antimicrobial and antioxidant properties [123], making them more effective in applications such as food preservation and medical treatments. The encapsulation process protects these compounds from degradation and allows for sustained release, ensuring long-lasting antimicrobial effects. Similarly, rutin and phenolic compounds derived from green propolis exhibit improved antibacterial and antioxidant activity when encapsulated [147]. This not only increases their ability to combat pathogens but also extends their efficacy, reducing the need for frequent reapplication. Overall, encapsulation significantly improves the antimicrobial performance of these bioactive compounds, making them more suitable for use in diverse applications, from food safety to wound care and infection control.

#### 5.2.6. Reduced Toxicity of Bioactive Compounds

Encapsulation plays a crucial role in reducing the potential toxicity of high-dose or potent compounds, ensuring their safe use without compromising their therapeutic benefits [175]. Many bioactive compounds, when used at high concentrations, can have toxic effects on cells and tissues. However, by encapsulating these compounds, their release is controlled, reducing the risk of toxicity while still maintaining their desired activity. For instance, vanillin and berberine, both known for their antimicrobial properties [148, 158], can be cytotoxic at higher concentrations. Encapsulation mitigates this toxicity by controlling the rate and site of release, allowing these compounds to retain their antimicrobial properties without causing harm to healthy cells. Similarly, Buriti oil and ginseng extract, both rich in bioactive compounds, are known to have therapeutic effects [152, 161], but their concentrated forms can cause irritation or adverse reactions if not properly managed. Encapsulation ensures the safe delivery of these oils and extracts by protecting them from degradation and controlling their release, thus minimizing the risk of toxicity. This makes encapsulated formulations of these compounds a safer and more effective option for a wide range of applications, from medicinal to cosmetic products [170].

#### 5.2.7. Multicompound Encapsulation for Enhanced Therapeutic Efficacy

Encapsulation enables the combination of multiple bioactive compounds into a single formulation, allowing for synergistic effects that enhance therapeutic outcomes [176]. This multifunctionality is particularly useful in combination therapies, where different compounds work together to target multiple pathways or mechanisms of action, improving the overall efficacy of treatment [177]. For example, coencapsulating ceftazidime and tobramycin in zein NPs coated with chitosan has shown stronger antibacterial and antibiofilm effects than using the drugs separately. These NPs offer a promising solution for treating infections caused by antibiotic-resistant and biofilm-forming bacteria like *P. aeruginosa* and *K. pneumoniae* [137]. By codelivering bioactive compounds, encapsulation enhances their therapeutic potential, providing a holistic approach to treating complex health issues. This multifunctional aspect of encapsulation makes it a promising strategy for the development of advanced combination therapies, where different bioactive agents can be delivered simultaneously, maximizing their synergistic effects and improving patient outcomes [177].

Biobased NPs provide an efficient and ecofriendly means of delivering antimicrobial agents, enhancing their stability, bioavailability, and therapeutic efficacy. Their biocompatibility and biodegradability make them highly suitable for a wide range of applications, including medical treatments, food preservation, and packaging, where effective bacterial control is essential [12]. These findings highlight the promising potential of biobased NPs as innovative solutions for combating microbial threats in various industries.

However, assessing their stability over time, temperature, and pH is crucial for their successful use in antimicrobial treatments. Stability determines how well these NPs retain their structure, functionality, and antimicrobial activity under various environmental and physiological conditions [178]. If NPs degrade, aggregate, or lose their functional integrity due to changes in storage time, temperature fluctuations, or varying pH levels, such as those found in different parts of the human body or in external environments, their therapeutic efficacy may be compromised [179]. Moreover, instability can lead to inconsistent dosing, reduced bioavailability, or unintended interactions with healthy tissues or the environment [180]. Therefore, ensuring that biobased organic NPs maintain their physicochemical properties across a range of conditions is essential for their reliable performance in disease management.

## 6. Challenges of Using Biobased Organic NPs and Possible Safety Measures

Biobased organic NPs offer a range of significant advantages, including biodegradability, biocompatibility, and sustainability, which make them highly attractive for diverse applications in medicine, food preservation, and packaging. Despite these benefits, their widespread use is accompanied by several challenges that must be addressed to fully realize their potential.

One of the primary concerns is the stability of biobased NPs under varying environmental conditions. These NPs can be sensitive to factors such as pH, temperature, and humidity, which can compromise their structural integrity and, consequently, their performance [181]. For example, chitosan-based NPs are known to degrade in different environments [182], which may limit their use in some therapeutic or preservation applications. While biodegradability is an advantageous feature, the rate at which biobased NPs degrade is influenced by factors like particle size, polymer composition, and environmental conditions. For instance, chitosan NPs tend to degrade faster in acidic conditions but may persist longer in neutral or alkaline environments [183], potentially raising concerns about their environmental impact and persistence in ecosystems.

Biocompatibility is another key strength of biobased NPs, as natural polymer-based NPs (such as those made from chitosan, cellulose, and starch) are generally considered safer and less toxic than their synthetic counterparts. Chitosan NPs, for example, have demonstrated low toxicity, making them widely used in drug delivery systems without causing significant adverse effects [184]. However, potential toxicity risks cannot be completely excluded. The degradation products of biobased NPs, such as lignin or chitosan, could accumulate in tissues over time, potentially triggering inflammation or other harmful reactions. Furthermore, the NP characteristics—such as size, shape, and surface charge—can influence how these particles interact with biological systems, sometimes leading to unexpected toxicity [185].

Another challenge involves achieving precise control over the release rates of drugs encapsulated within biobased NPs. Variations in NP size, shape, and surface properties can lead to inconsistencies in the release profiles, which may diminish the therapeutic efficacy of the encapsulated compounds [186]. Long-term use of drug-loaded biobased NPs also raises concerns about the potential for accumulation in tissues and the subsequent risk of side effects or toxicity.

From an environmental perspective, biobased NPs are generally considered less harmful than synthetic NPs. However, the large-scale release of these NPs still poses potential risks [187]. For instance, when biobased NPs are released into ecosystems in high concentrations, they could disrupt aquatic or soil organisms, affecting microbial populations and ecosystem health. While these NPs are biodegradable, their slow degradation in certain environments or during large-scale disposal could lead to unintended ecological consequences. Therefore, proper waste management practices are crucial to mitigate environmental hazards.

In conclusion, while biobased organic NPs hold great promise in various applications, it is essential to address challenges related to their stability, controlled drug release, potential toxicity, and environmental impact. By focusing on these issues, we can better harness the full potential of biobased NPs, particularly in antimicrobial and therapeutic fields, while ensuring their safety for both human health and the environment.

## 7. Conclusion and Future Perspectives

Biobased organic NPs offer a promising solution to fight microbial threats, including drug-resistant pathogens. Their small size, diverse shapes, adjustable surface charges, and stability enable effective interactions with microbes, providing strong antimicrobial activity. When used as drug delivery systems, NPs improve treatment by enhancing stability, bioavailability, and targeted release of compounds, while minimizing toxicity. This review emphasizes the potential of biobased NPs in addressing AMR and improving safety in healthcare, food preservation, and environmental protection. However, challenges remain for future research. These include optimizing NPs' size, shape, surface charge, and stability to improve their effectiveness against various pathogens, including drug-resistant strains. Understanding how NPs interact with microbial cells is crucial for improving their antimicrobial action and reducing resistance risks. Equally important is the advancement of safe, cost-effective, and environmentally friendly synthesis methods using renewable resources. Thorough safety evaluations are also critical to ensure their biocompatibility and long-term application.

Future research should prioritize the rational design of multifunctional NPs, deeper insights into their biological interactions, and the development of regulatory frameworks to guide their safe and effective use in sustainable disease management strategies.

## Figures and Tables

**Table 1:** Characteristics, stability, and antimicrobial properties of naturally derived organic NPs.

**NPs**	**Size**	**Shape**	**Surface charge**	**Stability**	**Pathogen**	**Effect**	**Ref**
Chitosan NPs	103 nm	Nearly spherical (with circularity of 0.967)	14.9 ± 3.1 mV	Stable within a 1-month period in a water stock solution	*P. aeruginosa*	Reduced biofilm development by 50% at concentrations ranging from 80 to 160 mg L^−1^	[65]
	80–364.4 nm	—	40 ± 3 mV (pH 4)	Smaller NPs remained stable with sizes of 87.3 ± 1.1 and 146.0 ± 8.0 nm, while larger ones increased significantly to 219.2 ± 10.1 and 274.7 ± 20.7 nm after 1 month at 4°C–8°C.	*Mycobacterium tuberculosis*, *E. coli*, *S. aureus*, *Enterococcus faecalis*, and *C. albicans.*	Chitosan NPs showed antimicrobial activity against all tested microorganisms. They effectively inhibited *M. tuberculosis* at 300 *μ*g (116.6 nm) and 400 *μ*g (364.4 nm), with the strongest effect against *E. faecalis* (200–280 nm).	[17]
	28.5–51.4 nm	Spherical	—	—	*S. aureus* and *E. coli*	Plasticized starch (PS) film loaded with chitosan NPs inhibited bacteria growth than neat PS film, with 100% reduction in colony-forming units against *S. aureus*	[66]
	33.1 nm	Nearly spherical	13.8 mV	—	*Helicobacter pylori*	NPs exhibited strong COX-2 inhibition (IC50 4.5 *μ*g/mL), exceeding indomethacin (0.08 *μ*g/mL) and celecoxib (0.79 *μ*g/mL) in effectiveness.	[67]

Chitosan-Schiff base 1 NPs (base 1: 2-(4-formylphenoxy)-N-phenylacetamide)	29.1 nm	Nearly spherical	12.0 mV	—	*H. pylori*	NPs exhibited strong COX-2 inhibition (IC50 4.5 *μ*g/mL), exceeding indomethacin (0.08 *μ*g/mL) and celecoxib (0.79 *μ*g/mL) in effectiveness.	[67]

Chitosan-Schiff base 2 NPs (base 2: N-(4-bromophenyl)-2-(4-formylphenoxy) acetamide)	13.3 nm	Nearly spherical	18.9 mV (positive)	—			

Nanocellulose and cellulose nanocrystals (CNC)	50 nm	Spherical shape	—	—	*S. aureus*, *Klebsiella pneumonia*, *E. coli*	The antimicrobial effect of nanocellulose on the tested bacteria was found to be superior to that of the antibiotics assessed in the study.	[68]
	Length 150 nm	Acicular and rod shaped	—	—	*P. syringae*	Weakly inhibited cell growth, induced bacterial aggregation, and inhibited biofilm production	[69]
	157.6 nm (DLS)	Cylindrical	−15.5 mV	—	*P. syringae*	Pretreating *Arabidopsis thaliana* with cellulose nanocrystals reduced infection levels caused by *P. syringae* by up to 65%.	[70]
	Width 5.2 nm and length 144 nm	Rod shaped	—	—	*E. coli*	Surfaces coated with CNCs were capable of inactivating around 90% of the attached *E. coli* cells.	[71]
	Length 81 and 93 nm (width 10 nm)	Needle-like and rod shaped	—	—	*P. savastanoi*	CNCs showed an interesting in vitro effect in inhibiting bacterial growth and bacterial biofilm formation	[72]

Lignin NPs (LigNPs)	292 nm	—	−34.4 ± 0.4 mV	NPs stable even after 6 months of storage at 4°C	*S. aureus*, *Bacillus cereus*, *P. aerugiosa*, and *E. coli*	Plant-derived polyphenol tannic acid-functionalized lignin NPs (PheLigNPs) showed superior antibacterial activity over nonfunctionalized (LigNPs) or bulk forms, due to higher phenolic content and nanoscale structure.	[48]
	143–151 nm.	—	−23.8 and −25.8 mV (negative surface charge)	NPs very stable in pure water	*B. cereus*, *S. aureus*, *S. sciuri*, *Salmonella typhi*, *S. enterica*, *E. coli*, *and A. favus*, *A. ochraceus*, *A. niger*, *Fusarium proliferatum*, and *Penicillium verrucosum*	All the studied microorganisms exhibited growth inhibition, with lignin nanoparticle treatments having a more significant impact on bacteria than on fungi.	[18]
	Range 40–300 nm (average 120 ± 18 nm)	—	—	—	*Colletotrichum gloeosporioides* and *Lasiodiplodia theobromae*	Presence of LigNPs significantly enhanced the growth inhibition of the polybutylene succinate/thymol composite.	[73]
	40–300 nm	Spherical	—	—	*S. aureus* and *E. coli*	At a 5 mg/mL concentration, LigNPs completely inhibited *S. aureus* and *E. coli*. LigNPs first disrupt the bacterial cell structure externally, then enter the cells, affecting protein synthesis and exerting antibacterial action.	[46]
	25–80 nm	—	−30.0 mV	Stable for more than 1 year in an aqueous environment at 5°C	*P. syringae, Xanthomonas axonopodis*, and *X. arboricola*	LigNPs were effective antibacterial agents against the tested bacteria.	[74]
	80 nm	Spherical	−29 ± 4 mV	—	*B. subtilis* and *Lactobacillus fermentum*	LigNPs have concentration-dependent toxicity to Gram-positive bacteria	[75]

Curcumin NPs	10–30 nm	—	—	—	Methicillin-resistant *S. aureus* (MRSA)	Curcumin NPs showed antibacterial activity against the MRSA strain, with the average minimum inhibitory concentration (MIC) for nanocurcumin calculated at 480.77 *μ*g/mL.	[76]
	80 ± 2 nm	Spherical	4.5 mV	Curcumin NPs powder had high physical stability	*S. aureus*, *E. coli*, and *B. cereus*	Curcumin NPs demonstrated significantly enhanced antibacterial activity in vitro. At a concentration of 10 *μ*g g ^−1^, Curcumin NPs were more effective in reducing microbial load in chicken fingers.	[77]
	9 nm and 18 nm	Spherical	—	—	*C. glabrata* and *A. niger*	Curcumin NPs (9 and 18 nm) exhibited strong antimicrobial activity against *C. glabrata* and *A. niger*, with the 9 nm NPs demonstrating superior effectiveness compared to the 18 nm particles.	[78]
	44 ± 8 nm average size	Rounded shape	43 ± 4 mV	—	*E. coli*, *S. typhimurium*, *Yersinia enterocolitica*, *S. aureus*, *B. cereus*, *A. niger*, *A flavus*, *Penicillium expansum*, and *C. albicans.*	Curcumin NPs demonstrated strong antioxidant activity (IC50 of 1550 *μ*g/mL) and exhibited antimicrobial properties against the tested bacteria and fungi in a dose-dependent manner.	[79]
	85 nm	Nearly spherical	—		*Streptococcus mutans*	All resin samples containing varying percentages of curcumin NPs exhibited antimicrobial properties against *S. mutans*.	[80]
	35.96 nm	—	—		*MRSA*, *S. epidermidis*, *L. innocua*, *P. aeruginosa*, *E. coli*, *S. typhi*, *A. oryzae*, and *Rhodotorula glutinis*	Curcumin NPs exhibited significantly enhanced antimicrobial activity compared to regular curcumin, attributed to their improved solubility, smaller size, shape, and optimized surface properties.	[81]

Curcumin, nisin-pectin NP composite	143 nm	—	−33.0 mV	—	*S. aureus* and *E. coli*	Chitosan/zein bilayer films with the nanoparticle composite were more effective against *S. aureus* than *E. coli*.	[82]

**Table 2:** Antimicrobial and antibiofilm activities of biobased organic NPs loaded with various drugs and materials against pathogenic microorganisms.

**NPs**	**Drug/material loaded**	**Pathogen**	**Effect**	**Ref**
Zein (Z) NPs (coated with chitosan)	Ceftazidime/Tobramycin	*P. aeruginosa* and *K. pneumoniae*	Coencapsulation formulation demonstrated superior antibacterial and antibiofilm activities compared to the individual formulations.	[137]

Zein (Z) NPs coated with chitosan/alginate (Alg)	Tea polyphenols and epigallocatechin gallate	*Vibrio anguillarum*, *V. alginolyticus*, *Photobacterium damselae*, *P. anguilliseptica*, and *S. iniae*	The Alg/chitosan-ZNPs formulations demonstrated strong growth inhibition, with over 90% inhibition observed against *P. damselae*	[138]

Chitosan NPs	Rutin	*P. aeruginosa*	Reduced *P. aeruginosa* motility, inhibited biofilm formation, and lowered pyocyanin and proteolytic activity	[139]
	Carvacrol	*E. coli* and *S. aureus*	High antibacterial performance against both *E. coli* and *S. aureus*, being more effective against *S. aureus*.	[140]
	Methicillin-resistant *S. aureus* (MRSA) phage	*—*	Encapsulating the MRSA phage with chitosan NPs enhanced its lytic activity and resistance to harsh conditions.	[141]
	*Trans*-resveratrol	*H. pylori*	Resveratrol encapsulated chitosan NPs inhibited bacterial growth, reducing the minimum inhibitory/bactericidal concentrations (MIC/MBC) by over 30 times compared to free resveratrol, eradicating biofilm, and lowering bacterial load	[142]
	*Zingiber officinale* essential oil	*E. coli*, *S. aureus, C. krusei* and *C. paraphimosis*	The antibacterial efficacy of chitosan NPs was determined, showing IC50 values of 661 *μ*g/mL against *E. coli* and 462 *μ*g/mL against *S. aureus*. Additionally, these NPs exhibited antifungal activity against *C. krusei* with a MIC of 4.25 *μ*g/mL and *C. parapsilosis* with a MIC of 8.5 *μ*g/mL.	[143]

Chitosan/sodium deoxycholic acid (SDC) nanoplexes	Ciprofloxacin	*P. aeruginosa* and *S. aureus*	The antibacterial and antibiofilm activities of ciprofloxacin against bacterial strains were significantly enhanced when loaded into chitosan/SDC NPs.	[144]

Cellulose nanocrystals	Chitosan and gallic acid	*P.* pathogens (*P. syringae* and *P. savastanoi*)	The nanostructured formulation inhibited ≤ 80% of the *pseudomonas* pathogens	[123]
	Nisin	*L. rhamnosus* and *Leuconostoc mesenteroides*	Microbial inactivation was achieved by nanocrystals loaded with 2.0 and 2.5 mg/mL nisin.	[124]

Starch nanofibers	Carvacrol@casein NPs	*B. cereus*	Inhibited the growth of *B. cereus*	[145]

Starch NPs	Triphala churna (herbal drug)	*S. typhi* and *Shigella dysenteriae*	The starch NPs encapsulating Triphala Churna demonstrated strong antimicrobial activity against *S. dysenteriae*, *S. typhi*, and *S. aureus*, as well as notable antibiofilm activity against *ATCC MRSA 33591* and the *N7 clinical strain*.	[146]
	Phenolic compounds (PCs) from green propolis (*p*-coumaric acid, rutin, kaempferol, and quercetin)	*Listeria monocytogenes*	Positive antimicrobial activity observed, proving effectiveness of PC loaded on starch NPs in inhibiting *L. monocytogenes*.	[147]
	Vanillin	*E. coli*	The antimicrobial activity of vanillin-loaded starch NPs was evaluated against *E. coli* with satisfactory results.	[148]

Starch-based nanocapsules	Curcumin	*E. coli* and *S. aureus*	Nanocapsules loaded with curcumin exhibited strong antibacterial properties, particularly against *S. aureus*. In contrast, carriers without curcumin showed no antibacterial effect.	[149]

Phospholipid nanofibers	Eugenol@cationic starch NPs	*B. cereus*	The population of *B. cereus* decreased by 98.98%	[150]

Lignin NPs (LigNPS)	*Ocimum basilicum* extract (OB) and *Lagenaria siceraria* seed oil (LS)	*E. coli*, *E. faecalis*, *K. pneumoniae*, *S. aureus*, *S. enterica*, *Trichophyton mentagrophytes*, *T. rubrum*, and *Microsporum canis.*	Both OB-LigNPs and LS-LigNPs exhibited strong antimicrobial activity against multidrug-resistant bacteria and fungi.	[151]
	Ginseng (Gn)	*E. coli*, *B. megaterium*, and *C. tropicalis*	The developed GnLigNPs demonstrated significant antimicrobial activity against *E. coli*, *B. megaterium*, and *C. tropicalis*, surpassing the effects of both ginseng and bare LigNPs.	[152]
	Curcumin	*S. aureus*, *E. coli*, and *P. aeruginosa*	Curcumin-modified LigNPs exhibited significantly enhanced antibacterial activity, with a notably lower minimum inhibitory concentration (MIC) compared to either LigNPs or curcumin alone.	[153]

Pectin NPs	Nisin	*Arthrobacter* sp., *B. subtilis*, *E. coli*, and *Klebsiella sp.*	Nisin-loaded pectin NPs exhibited greater antibacterial activity compared to sodium benzoate against all bacteria tested in the experiment.	[154]
		*S. typhimurium and L. innocua*	The combined effect of pulsed electric field and nisin-loaded pectin NPs increased the susceptibility of bacteria	[155]
	Ceftizoxime	*B. cereus*, *B. polymyxa*, *Enterobacter aerogenes*, and *P. aeruginosa.*	The nanoformulation exhibited sustained drug release, and the drug-loaded NPs demonstrated strong antimicrobial activity against all tested microorganisms.	[156]

Alginate (Alg) NPs	*Cuminum cyminum* (CC) and *Zataria multiflora* (ZM) essential oils	*E. coli*, *P. aeruginosa*, and *S. aureus*	Alginate treatment alone showed negligible antibacterial activity against all bacterial strains, highlighting the carrier material's lack of inherent antimicrobial properties. In contrast, Alg-ZM exhibited significantly stronger antibacterial effectiveness compared to Alg-CC against all tested bacteria.	[157]
	Berberine	*B. subtilis*, *K. pneumonia*, *E. coli*, and *P. aeruginosa.*	Berberine loaded sodium alginate NPs show higher entrapment efficiency and enhanced antibacterial activity.	[158]

Chitosan-sodium alginate NPs	Rifaximin	*B. haynesii*, *P. aeruginosa*, and *E. coli*	Rifaximin@chitosan/Alg-NPs demonstrated excellent antibacterial activity against *E. coli*, *P. aeruginosa*, and *B. haynesii*, with inhibition zones of 24, 30, and 34 mm, respectively.	[159]
	LysMR-5 (an endolysin)	*S. aureus* and *S. epidermidis*	Alg-chitosanNPs exhibited antibacterial effects against *S. aureus*, with an increase in bactericidal activity upon loading with LysMR-5.	[160]

Gelatin NPs	Buriti (Mauritia flexuosa) oil	*P. aeruginosa*, *K. pneumonia*, and *S. aureus*	The antimicrobial activity of the oil was significantly enhanced by 59%, 62%, and 43% against *P. aeruginosa*, *K. pneumoniae*, and *S. aureus*, respectively.	[161]

## Data Availability

The authors have nothing to report.
